# Case report: new development of fibrosing interstitial lung disease triggered by HIV-related pneumocystis pneumonia

**DOI:** 10.1186/s12890-019-0831-9

**Published:** 2019-03-18

**Authors:** Tetsuya Suzuki, Yukiko Shimoda, Katsuji Teruya, Hiroyuki Gatanaga, Yoshimi Kikuchi, Shinichi Oka, Koji Watanabe

**Affiliations:** 1 AIDS Clinical Center, National Center of Global Health and Medicine, 1-21-1 Toyama, Shinjuku-ku, Tokyo, 162-8655 Japan; 2 Department of respiratory medicine, National Center of Global Health and Medicine, Tokyo, Japan; 3 Center for AIDS Research, Kumamoto University, Kumamoto, Japan; 4 Disease Control and Prevention Center, National Center for Global Health and Medicine, Tokyo, Japan

**Keywords:** HIV, Pneumocystis pneumonia, Fibrosing interstitial lung disease, Pulmonary fibrosis, Interstitial pneumonia

## Abstract

**Background:**

Fibrosing interstitial lung disease is the poor prognostic non-infectious lung disease by unknown etiology. Here, we present one case developing interstitial pneumonia with fibrosis after treatment of pneumocystis pneumonia (PCP) in newly diagnosed HIV-1 infected case.

**Case presentation:**

A previously healthy 63-year old male was referred to our institute because of protracted dyspnea on effort in 2 weeks after pneumocystis pneumonia treatment. At referral, arterial blood oxygen pressure was within normal range (93.5 mmHg) at rest, but decreased rapidly 30 s after a slow walk (44.5 mmHg). Respiratory function tests showed severe restrictive ventilator impairment (vital capacity = 36.5%; forced expiratory volume in 1 s = 107.4%). Chest computed tomography showed severe fibrotic changes at bilateral basal parts and diffuse fibrotic changes in which PCP lesions were seen initially in previous images although β-D glucan was not elevated and *P. jirovecii* was not detected in saliva at referral. Other etiologies of fibrotic IP including infectious and/or autoimmune diseases were excluded by serology. Fibrotic lesion did not expand thereafter although it had not responded to the high-dose corticosteroid therapy.

**Conclusion:**

We report the first case of fibrosing interstitial lung disease triggered by HIV-related PCP.

## Background

Fibrosing interstitial lung disease is the poor prognostic non-infectious lung disease by unknown etiology. Here, we present one case developing interstitial pneumonia with fibrosis after treatment of pneumocystis pneumonia (PCP) in newly diagnosed HIV-1 infected case.

## Case report

A previously healthy Japanese 63-year-old male was referred to the AIDS Clinical Center, National Center for Global Health and Medicine (Tokyo, Japan; day 0) because of protracted dyspnea on effort (DOE) after pneumocystis pneumonia (PCP) treatment.

**Fig. 1:**
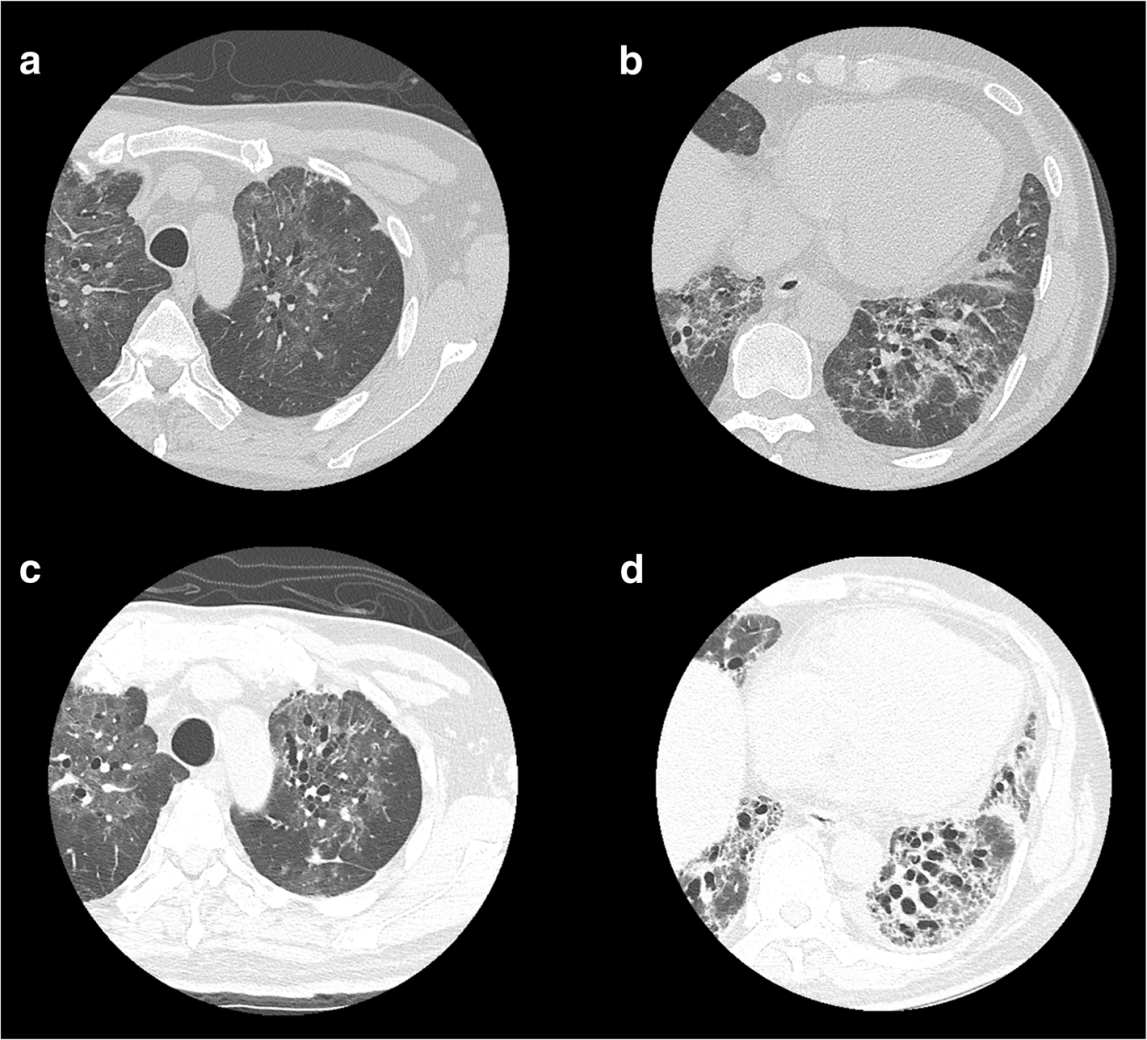
High-resolution computed tomography of the chest before (**a** and **b**, day − 56) and after (**c** and **d**, day 14) treatment of pneumocystis pneumonia

Fifty-seven days before referral (day − 57), he was admitted to a local hospital for progressive dyspnea of one month duration with diffuse interstitial infiltration in bilateral lung fields (chest computed tomography (CT), Fig. 1a, b). Human immunodeficiency virus (HIV) infection was first pointed out upon initial blood examination, and the non-acute phase of HIV-1 infection was confirmed by western blotting. Cluster of differentiation-4 counts and HIV-RNA loads were 45/μL (7.3%) and 56,000 copies/mL, respectively. Bronchoscopy identified *Pneumocystis jirovecii* in bronchoalveolar lavage fluid, and levels of β-D glucan in serum was increased (> 300 pg/mL) at that time point. With a diagnosis of HIV-related PCP, trimethoprim-sulfamethoxazole (TMP-SMX) was initiated with corticosteroids at that hospital (day − 47). Hypoxia under rest was improved rapidly, but DOE remained 2 weeks after completion of PCP treatment (day − 12). He was referred to our hospital for the further examination and treatment (day 0). Physical examination revealed “Velcro rales” in bilateral lower back auscultation, whereas no other abnormalities were identified by a review of systems (including neurologic examination). Arterial blood oxygen pressure was within normal range (93.5 mmHg) at rest, but decreased rapidly 30 s after a slow walk (44.5 mmHg). Respiratory function tests showed severe restrictive ventilator impairment (vital capacity = 36.5%; forced expiratory volume in 1 s = 107.4%). Re-examination of chest CT showed severe fibrotic changes at bilateral basal parts and diffuse fibrotic changes (Fig. 1c, d) in which PCP lesions were seen initially in previous images. Levels of SP-D (214.2 ng/mL), KL-6 (2249 IU/mL) and lactate dehydrogenase (234 IU/L) were increased, but β-D glucan was not elevated (14.8 pg/mL) and *P. jirovecii* was not detected in saliva at referral. Cytomegalovirus DNA in plasma and surrogate markers of autoimmune diseases (anti-centromere antibody, anti-Jo-1 antibody, c-ANCA, p-ANCA, anti-nuclear antibody, anti-RNP antibody, anti-SS-A antibody, anti-SS-B antibody, anti-Scl-70 antibody, anti-CCP antibody) was negative. The drug-induced lymphocyte stimulation test (DLST) against TMP-SMX was negative, but we changed secondary prophylaxis of PCP to atovaquone. We initiated combination antiretroviral therapy (cART) comprising dolutegravir, tenofovir alafenamide and emtricitabine at day 2, and added tacrolimus at day 16, but respiratory function and imaging findings were not improved. Corticosteroids (methylprednisolone [1000 mg/day, p.o.] for 3 days followed by prednisolone [1 mg/kg/day, p.o.]) were re-initiated at day 37. However, we stopped corticosteroid therapy because it caused mediastinal emphysema without eliciting any positive effects upon lung fibrosis. We started nintedanib at day 48, and are following up symptoms carefully. Oxygen requirement until the last follow-up date (day 120) was unchanged, that is 2 L/min at rest or 4 L/min at light labor.

## Discussion and conclusions

Fibrosing interstitial lung disease, especially non-specific interstitial pneumonia (NSIP) and lymphoid interstitial pneumonitis, is more common among HIV-positive individuals than among HIV-negative ones, and cART could be effective in some cases [1, 2]. Drugs and autoimmune diseases can also induce acute lung damage that mimics NSIP [3, 4]. In our case, PCP was diagnosed initially by identification of *P. jirovecii* at a local hospital. Thereafter, fibrotic changes progressed despite successful treatment for PCP. Organ damage other than bilateral lung fields was not identified by imaging (contrast-enhanced CT from the neck to the abdomen; magnetic resonance imaging of the brain) or physical examination. Serological tests for autoimmune diseases were within normal ranges. We cannot completely exclude the possibility that drugs induced interstitial pneumonia, but DLST against TMP-SMX was negative, and fibrosis was not improved by cessation of treatment with TMP-SMX or corticosteroids. Furthermore, fibrotic changes spread in accordance with those seen for PCP lesions with severe restrictive ventilatory impairment. cART or corticosteroids had no beneficial effects on fibrosis. We diagnosed fibrosing interstitial lung disease by PCP from these clinical courses even though a pathologic diagnosis using expanded lung biopsy samples under surgery was not made due to severe restrictive ventilatory impairment. Another possible etiology was that PCP induced acute exacerbations of undiagnosed idiopathic pulmonary fibrosis which was present asymptomatically for a long time before PCP. However, our patient underwent annual health checks, and we confirmed that chest radiography was normal about one year before PCP onset. Furthermore, pulmonary fibrosis was limited within PCP lesions and did not progress after completion of PCP treatment.

In conclusion, we report the first case of fibrosing interstitial lung disease triggered by HIV-related PCP.
